# Platelet-rich plasma combined with allograft to treat osteochondritis dissecans of the knee: a case report

**DOI:** 10.1186/s13256-019-2027-6

**Published:** 2019-04-24

**Authors:** Mikel Sánchez, Diego Delgado, Ane Garate, Pello Sánchez, Sabino Padilla, Juan Azofra

**Affiliations:** 1 Arthroscopic Surgery Unit, Hospital Vithas San José, C/ Beato Tomás de Zumarraga 10, 01008 Vitoria-Gasteiz, Spain; 2 Advanced Biological Therapy Unit, Arthroscopic Surgery Unit, Hospital Vithas San Jose, Vitoria-Gasteiz, Spain; 3 University Institute for Regenerative Medicine & Oral Implantology – UIRMI (UPV/EHU-Fundacion Eduardo Anitua), Vitoria-Gasteiz, Spain

**Keywords:** Platelet-rich plasma, Osteochondritis dissecans, Allograft, Knee surgery, Tissue repair

## Abstract

**Background:**

Osteochondritis dissecans of the knee is a prevalent pathology in young, active people that is brought about by either traumatic, developmental, or iatrogenic etiologies.

**Case presentation:**

A 40-year-old Caucasian man reporting pain, swelling, and functional reduction was evaluated and diagnosed with internal condyle osteochondritis dissecans of the knee. Harnessing the trophic, chondroprotective, anti-inflammatory, and immunomodulatory properties of platelet-rich plasma, we carried out a knee open-sky surgical technique in which we combined autologous therapy with osteochondral allograft to treat the focal, large, and deep traumatic-iatrogenic osteochondritis dissecans of the knee. The axial computed tomographic scan taken 1 year after surgery revealed an area of abnormal signal intensity that was reduced on a computed tomographic scan 2 years later. The computed tomographic scan obtained 2 years later and the magnetic resonance imaging scan 3 years later also showed a clear reattachment and incorporation of the graft. Seven years after the surgery, the patient resumed his daily routine without any recurrent symptoms.

**Conclusion:**

Platelet-rich plasma application in osteochondral allograft implantation open surgery could enhance the healing process of medial condyle osteochondritis dissecans of the knee.

## Background

Osteochondritis dissecans of the knee (OCD) is a prevalent pathology in young, active people that is brought about by traumatic, developmental, or iatrogenic etiologies, entailing devastating short- and long-term clinical consequences. Recent advances made in tissue engineering approaches for articular cartilage repair using allografts and/or growth factor-based therapies have shown promising results [1]. We aimed at combining platelet-rich plasma (PRP) with osteochondral allograft as a cartilage matrix scaffold to treat focal, large, and deep traumatic-iatrogenic OCD, all of which were associated with a rehabilitation program,

## Case presentation

A 40-year-old Caucasian man reporting pain, swelling, and functional reduction without severe effects on the range of motion was evaluated. Fourteen years ago, the patient had been diagnosed with medial femoral condyle OCD, which was treated under arthroscopic osteosynthesis with three Herbert screws. Four years after the first intervention, and reporting unsatisfactory clinical progress, the patient underwent a valgus osteotomy of the tibia and an exploratory arthroscopy in which two of the three screws of the medial femoral condyle were removed. In spite of the latter treatment, the knee pain and swelling lingered, so we assessed the size of OCD by obtaining cartilage-specific axial computed tomographic (CT) scan sequences, which evinced a cartilage defect 1.83 cm deep and 1.52 cm wide in the medial femoral condyle associated with a mobile fragment. We made the decision to perform an open knee surgery using an osteochondral allograft (OCA) assisted with PRP.

PRP was prepared according to Endoret®(pgrf®) technology (BTI, Vitoria-Gasteiz, Spain) [2]. Before inducing anesthesia and starting prophylactic antibiotic treatment and saline, 80 ml of peripheral venous blood was withdrawn into 9-ml tubes containing 3.8% (wt/vol) sodium citrate as anticoagulant. Blood was centrifuged at 580 *g* for 8 min at room temperature. In each tube, the 2-ml plasma fraction located just above the sedimented red blood cells was collected in a tube without aspirating the buffy coat. This PRP contained 1.5 to 2.5 times the concentration of platelets compared with peripheral blood and an absence of erythrocytes and leukocytes. The activation of PRP was carried out by adding calcium chloride (10% wt/vol). Some of the liquid was incubated at 37 °C for 30 min in a glass dish, which allowed a fibrin membrane to form. The rest of the liquid PRP was infiltrated during surgical intervention as follows.

**Fig. 1:**
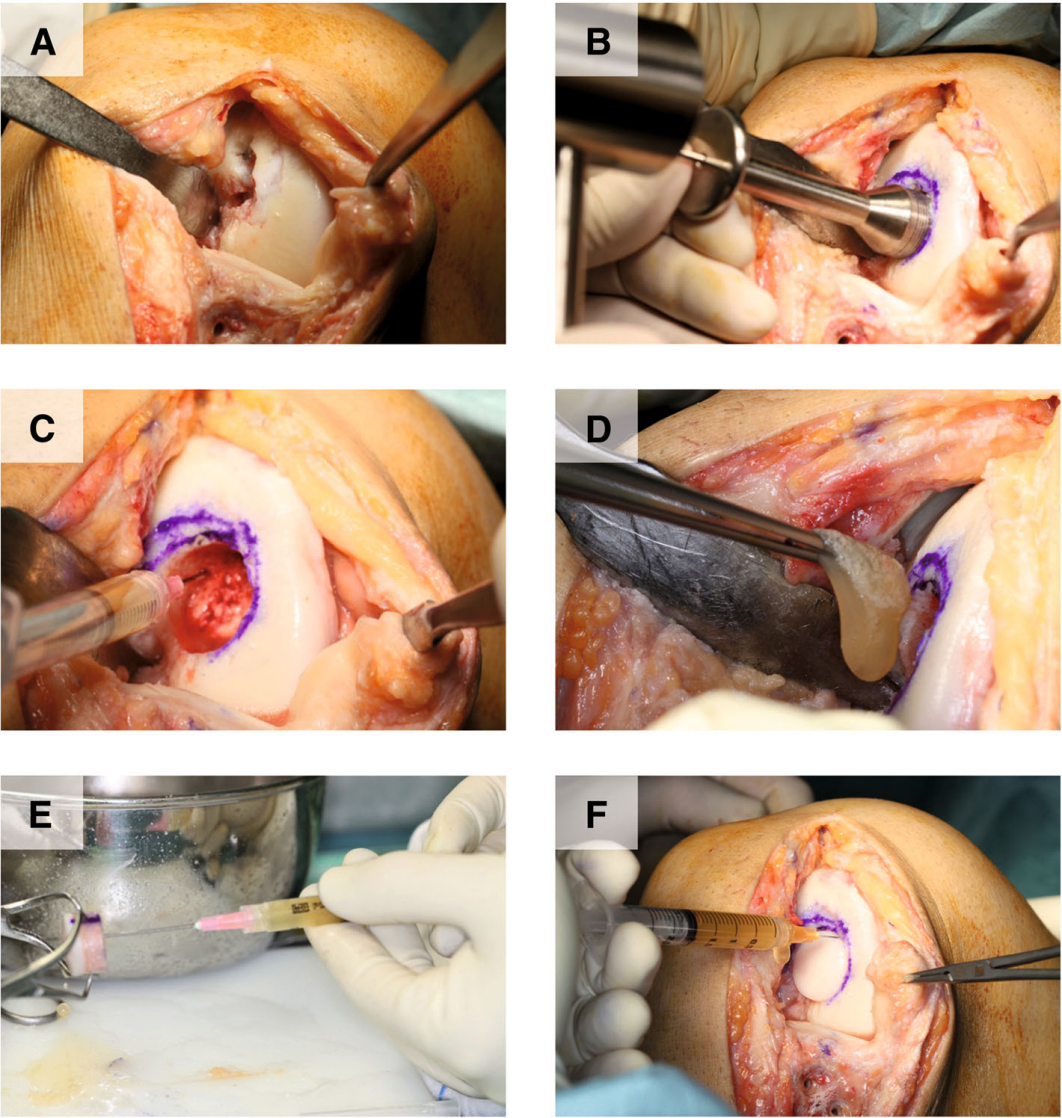
Different steps of osteochondral allograft implantation combined with platelet-rich plasma (PRP) to treat osteochondritis dissecans of the knee. A debridement, spongialization, and reaming of the osteochondral wound bed was performed (**a** and **b**). Pridie-type microfractures followed by infiltration of liquid PRP into subchondral bone were carried out (**c**). PRP membrane was placed into the wound bed (**d**). Next, the femoral plug osteochondral allograft was infiltrated with liquid PRP (**e**) and press-fit into the reamed area, sealing the interface around the allograft with liquid PRP (**f**)

An arthrotomy was performed following the previous incision made to conduct the valgus osteotomy. The plaque and screws of the valgus osteotomy were removed as well as the Herbert screw, which protruded from the hyaline cartilage. A debridement, spongialization, and reaming of the osteochondral wound bed was performed in order to achieve a defect of 2 cm in diameter as the allograft (Fig. 1a and b). Then, Pridie-type drilling microfractures followed by infiltration of 4–5 ml of activated PRP into subchondral bone (Fig. 1c) was carried out. Afterward, we placed the fibrin membrane obtained as described above into the wound bed (Fig. 1d). Once the 1.25-cm deep femoral plug OCA was infiltrated with PRP (Fig. 1e), we press-fit it into the reamed area and sealed the interface around the allograft with activated PRP (Fig. 1f). When surgery was completed, we carried out an intra-articular infiltration of 8 ml of activated PRP. A further three intra-articular infiltrations of 8 ml of activated PRP were conducted on a weekly schedule during the postoperative period on an outpatient basis. Assisted walking with crutches and a minimal initial load was recommended during the first 4 weeks postintervention. A rehabilitation program with passive mobility and avoiding axial movements was initiated 2 weeks after the surgery. After week 4, partial support and resistance-free cycling, together with swimming pool exercises, were allowed.

**Fig. 2:**
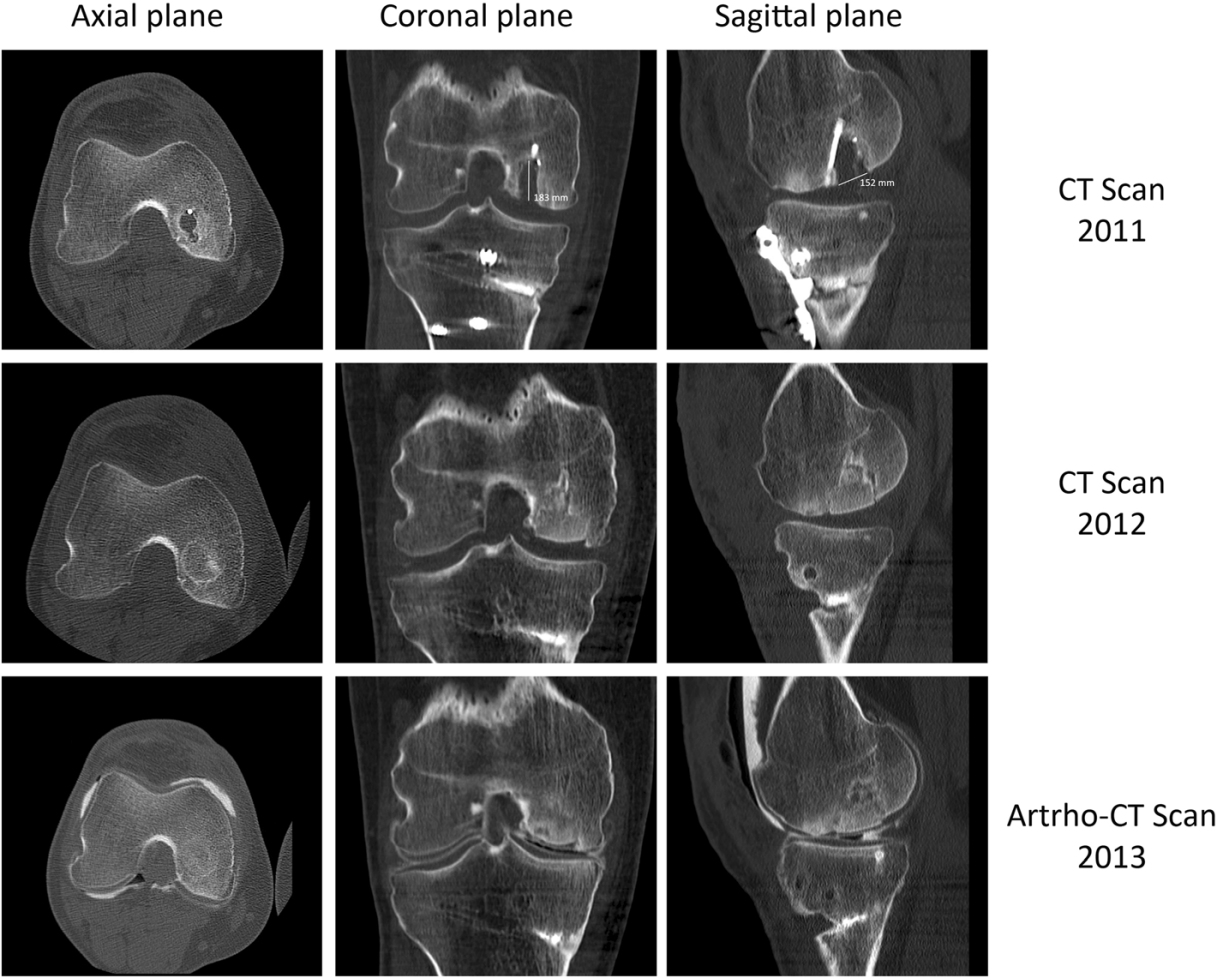
Axial computed tomographic (CT) scan and arthroscopic CT scan follow-up. Images obtained in 2011 show the defect in the femoral condyle where the osteochondral allograft was placed as can be observed in a CT scan obtained in 2012. In 2013, the graft evolved from an area of abnormal intensity signal to a satisfactory reattachment of the graft 2 years after the surgery

**Fig. 3:**
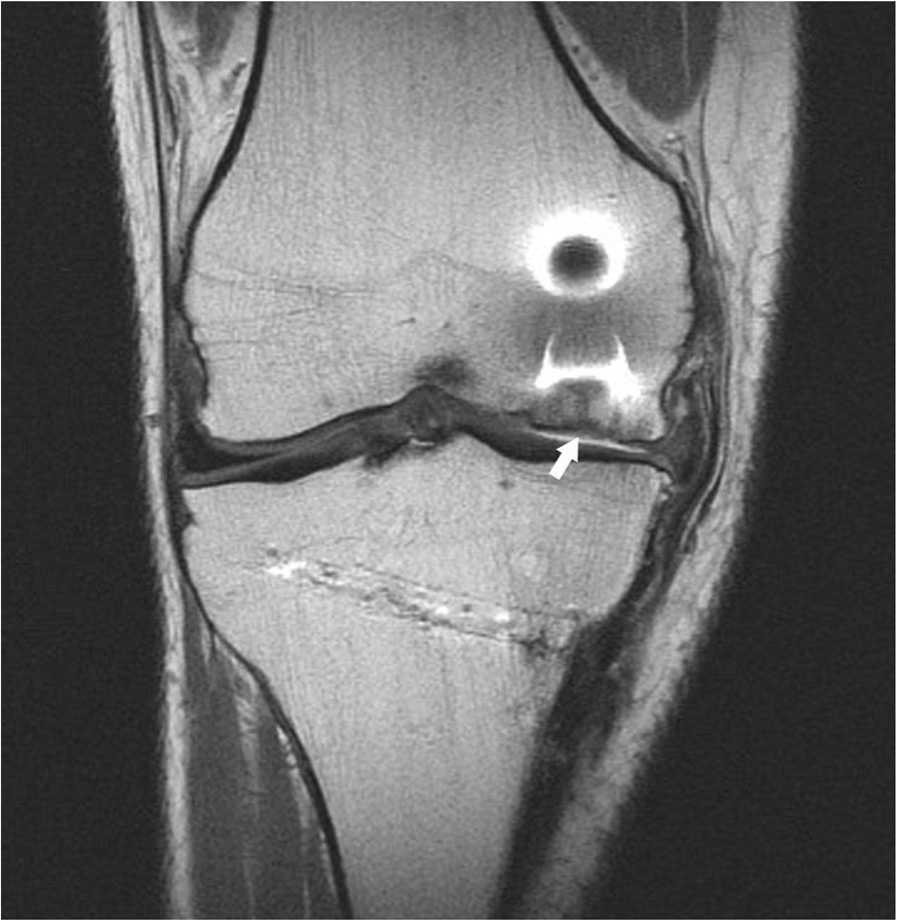
Magnetic resonance images obtained at follow-up. Images obtained in 2014 show the allograft integration into the medial condyle and the cartilage line under the graft (*white arrow*) 3 years after the intervention

The CT scan taken 1 year after the surgery revealed an area of abnormal signal intensity that was reduced in CT scans obtained 2 years later. In addition, the CT scan obtained 2 years later showed a clear reattachment and incorporation of the graft in an asymptomatic patient (Fig. 2). Magnetic resonance imaging (MRI) scans obtained 3 years after the surgery and examined by an experienced radiologist showed that the fragment remains well integrated, and the cartilage line under the graft was perceived (Fig. 3). Seven years after the surgery, the allograft was still incorporated into the joint without signs of failure. The patient resumed a daily routine without any recurrent symptoms of pain or swelling and with a suitable range of motion from 0 degrees of extension to 130 degrees of flexion.

## Discussion and conclusions

Although the use of several types of fixation tools, including screws and wires, have shown good results [3], this biologically based procedure combines the route from a mechanistic mode of intervention with a natural one by using an autologous therapeutic product.

The rationale for using PRP is that this autologous platelet concentrate forms an *in situ*-generated fibrin matrix delivery system and plays key roles in tissue regeneration and cartilage defect repair. In its form as a liquid-to-gel transition 3D injectable scaffold, PRP conveys many growth factors, cytokines, and chemokines contained in platelets, as well as fibrinogen and other plasmatic proteins, and gradually releases them into the injected area. Whether through one or a combined action of several growth factors, PRP has been proven to exert a multitarget trophic effect on synovial joint tissue cells and to improve osteochondral lesions [3–7].

During surgical technique, the subchondral bone is infiltrated with PRP in order to stimulate the crosstalk between subchondral bone and articular cartilage. In doing so, chemokines such as stromal cell-derived factor 1 and platelet factor 4 present within PRP might activate the migration of mesenchymal stem cells and chondrogenic progenitor cells from subchondral bone to homing the cartilage matrix allograft and even to modulate their differentiation into chondrogenic lineage by the presence of transforming growth factor-β [8, 9]. The MRI scan obtained 3 years after surgery suggests that these positive effects are related to cartilage tissue (Fig. 3). This migration would not take place in the presence of elevated concentration of proinflammatory cytokines [5, 9, 10].

The application of PRP is gaining ground within the therapeutic spectrum to enhance the otherwise limited repair capacity of the articular cartilage, based on an increasing body of evidence that comes primarily but not solely from basic science. First of note is a chondroprotective and anabolic effect through the induction of the synthesis of hyaluronic acid, proteoglycans, and collagen type II on synoviocytes and chondrocytes [4, 6]. Of the next significance is an anti-inflammatory and immunomodulatory effect produced by inhibiting the intracellular pathway nuclear factor-κB on chondrocytes and on macrophages, as well as by suppressing inflammatory mediators such as metalloproteinases [3, 5, 10, 11]. Third is the promotion of synoviocyte and chondrocyte viability and proliferation [5]. Further perspectives have been gained in the wake of excellent clinical outcomes obtained by the intraarticular injections of plasma rich in growth factors in knee and hip osteoarthritis [2], when we carried out the same protocol of three intraarticular infiltrations of PRP on a weekly basis to create a trophic intraarticular environment.

By using an OCA, the frequent outcome of donor site morbidity was circumvented, and furthermore, as revealed by the CT scan obtained at 2-year follow-up, the graft fit very well with the contour of the joint surface. In addition, this image as well as MRI revealed a satisfactory incorporation of the graft without separation of the fragment and the cartilage line. This together with the absence of symptoms 7 years after the surgery must be considered an excellent clinical result. Therefore, PRP application in an OCA implantation open surgery could enhance the healing process of internal condyle OCD.

## Abbreviations

CT: Computed tomography
MRI: Magnetic resonance imaging
OCA: Osteochondral allograft
OCD: Osteochondritis dissecans of the knee
MRI: Magnetic resonance imaging
PRP: Platelet-rich plasma
